# Diabetes Risk Score in a Young Student Population in Jordan: A Cross-Sectional Study

**DOI:** 10.1155/2017/8290710

**Published:** 2017-04-29

**Authors:** Abdel-Ellah Al-Shudifat, Amjad Al-Shdaifat, Ahmad Ali Al-Abdouh, Mohammad Ibrahim Aburoman, Sara Mohammad Otoum, Amro Ghaleb Sweedan, Ibrahim Khrais, Ibrahim Hisham Abdel-Hafez, Asgeir Johannessen

**Affiliations:** ^1^Faculty of Medicine, Hashemite University, Zarqa, Jordan; ^2^Centre for Imported and Tropical Diseases, Oslo University Hospital, Oslo, Norway

## Abstract

*Background*. The Middle East is the home to the most obese population in the world, and type 2 diabetes mellitus is endemic in the region. However, little is known about risk factors for diabetes in the younger age groups. *Methods*. The Finnish Diabetes Risk Score (FINDRISC) is a simple, validated tool to identify persons at risk of diabetes. We investigated students at Hashemite University in Jordan with FINDRISC and measured fasting plasma glucose in those who were categorized in the high-risk group. *Results*. Overall, 1821 students (881 [48.4%] female) were included in the study. Risk factors for diabetes were common: 422 (23.2%) were overweight or obese and 497 (27.3%) had central obesity. Using the FINDRISC score, 94 (5.2%) students were at moderate risk and 32 (1.8%) at high risk of diabetes. The mean FINDRISC score was significantly higher in men than women (5.9 versus 5.4; *p* = 0.002). Twenty-eight students in the high-risk group had a subsequent plasma glucose measurement, and 8 (29%) of them fulfilled the diagnostic criteria for diabetes. *Conclusions*. Risk factors for diabetes were common in a young student population in Jordan, suggesting that preventive measures should be initiated early in adulthood to turn the diabetes epidemic in the region.

## 1. Introduction

Diabetes mellitus is one of the most common chronic diseases today, both in developed and in developing countries. According to recent estimates of the World Health Organization (WHO), 422 million people in the world had diabetes in 2014, and the prevalence of people with diabetes is expected to double between the years 2000 and 2030 [1, 2].

The disease is characterized by a long period in a prediabetic state, with impaired fasting glucose, impaired glucose tolerance and, in many cases, the metabolic syndrome [3]. Advancing age, obesity, upper body fat distribution, inactivity, and a family history of diabetes are among the well-established risk factors for diabetes [4]. The Middle East has the highest prevalence of obesity and metabolic syndrome in the world and is expected to have the greatest increase in noncommunicable diseases and their risk factors in the near future [5–7].

Identifying individuals with undiagnosed type 2 diabetes mellitus (T2DM) is a public health priority, since the progression to complicated T2DM can be slowed or stopped with lifestyle modifications [8] or pharmacological interventions [9]. Based on 10-year prospective data on the incidence of T2DM in a population-based cohort, the Finnish Diabetes Risk Score (FINDRISC) was developed to identify subjects at high risk for future occurrence of T2DM. It is a simple, fast, inexpensive, noninvasive, and reliable tool to identify individuals at high risk for T2DM [10].

In order to plan effective prevention and control programs, the number of people at risk of future diabetes should be established. The previous studies from the Middle East have focused mainly on the adult population [11–13]. The younger generation, who grew up with internet and social media, might differ from their parents in terms of heath awareness, food habits, and other risk factors, but few studies have assessed risk factors for diabetes among the young population in the Middle East.

In the present study, therefore, we aimed to determine the prevalence of diabetes risk factors among young student population in Jordan.

## 2. Materials and Methods

### 2.1. Study Setting and Participants

This was a cross-sectional study conducted at Hashemite University in Zarqa, Jordan. Participants were recruited by convenience sampling among university students within a 2-month period (January to February 2014). Students were approached at canteens, lecture halls, and tutorial rooms whenever available. After verbal informed consent, the participants completed a self-administered paper survey. The questionnaire was an Arabic translation of the validated FINDRISC tool. The study was approved by the institutional review board (IRB) committee at Hashemite University.

### 2.2. Anthropometric Measurements

After completing the questionnaire, each student was subjected to weight and height measurements. Height was measured to the nearest 1 cm with participants in a standing position without shoes using a wall-fixed scaled meter. Body weight was measured to the nearest 0.1 kg, with minimal clothing and without shoes, using a portable electronic scale. Body mass index (BMI) was calculated as the ratio of weight in kilograms to the square of height in meters and was categorized according to the classification system established by the National Institutes of Health (NIH): normal body weight, 18.5–24.9; overweight, 25.0 to 29.9; and obese, >30.0 [14]. Waist circumference was measured midway between the lowest rib and the superior border of the iliac crest.

### 2.3. Blood Tests

Students who were classified to be at high risk of diabetes were invited to be measured for fasting blood glucose. Diabetes was diagnosed based on fasting plasma glucose in accordance with internationally recognized criteria: normal, <100 mg/dL; prediabetes, 100–125 mg/dL; and diabetes mellitus, ≥126 mg/dL [15].

### 2.4. Statistical Analysis

Descriptive data were expressed as frequency and percentage. We used chi-square tests to compare categorical variables and Student *t*-tests for continuous variables. SPSS version 23.0 software (SPSS Inc., Chicago, IL, USA) was used to analyse data, except for confidence intervals for proportions which were calculated with Open Epi version 3.01 [16]. All tests were two-sided and the level of significance was set at *p* < 0.05.

## 3. Results

**Figure 1:**
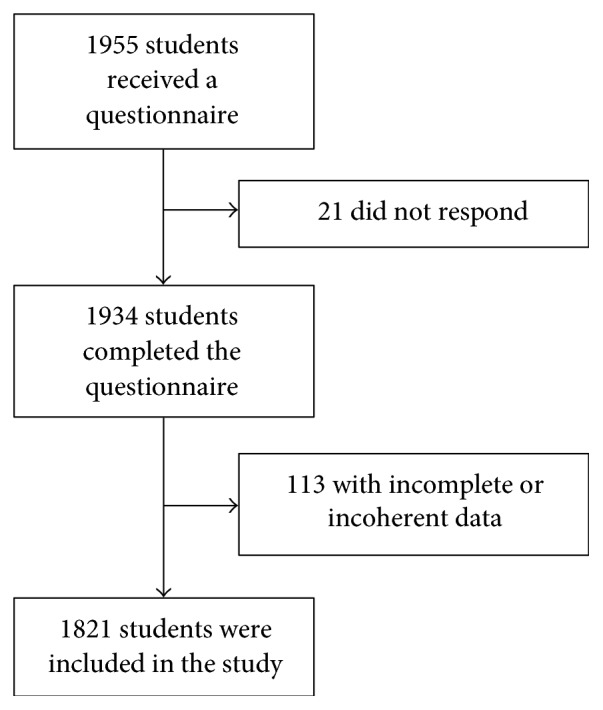
Profile of the inclusion and exclusion process.

Out of 1955 questionnaires distributed to students at Hashemite University, 1934 were returned and considered for the present study. Among them, 113 were excluded due to missing or incoherent information in the questionnaire. Thus, the final analysis included 1821 students, of whom 881 (48.4%) were female and 940 (51.6%) were male (Figure 1). Students ranged in age from 18 to 25 years.

Overweight was common in this young population; overall, 23.2% were either overweight (BMI 25.0–29.9 kg/m^2^) or obese (BMI ≥ 30 kg/m^2^). A significantly higher proportion of men were overweight and obese compared to that of women (Table 1). Similarly, a large proportion of study subjects had central obesity; 27.3% had a waist circumference above the FINDRISC threshold of 94 cm for men and 80 cm for women. About half of the participants (57.4%) reported regular physical activity, defined as a minimum of 30 minutes daily during either work or leisure time.

The majority of the study subjects reported that they had at least one first or second degree relative who had been diagnosed with diabetes. Overall, as many as 49.9% had a grandparent, uncle, aunt, or first cousin with diabetes, while 24.8% reported that they had a parent, sister, brother, or a child with diabetes. A total of 3.8% of study participants recalled that they had at least once tested positive for high blood sugar during health examination, illness, pregnancy, etc. Unsurprisingly, only 1.2% of these young students reported taking medication for high blood pressure.

With regard to the FINDRISC, 1218 (66.9%) students were found to have a score of less than 7, placing them at low risk of diabetes. Of study subjects with a score of 7 and more, 477 (26.2%) had a score between 7 and 11 corresponding to a slightly elevated risk, 94 (5.2%) had a score between 12 and 14 indicating a moderate risk, and 32 (1.8%) scored 15 or above which indicates a high risk of diabetes. Men had a significantly higher mean FINDRISC score than women (5.9 versus 5.4; *p* = 0.002) and were overrepresented in the groups with moderate or high risk of diabetes (Table 2).

Among 32 individuals who were categorized in the high-risk group (FINDRISC score ≥ 15), 28 were available for a subsequent fasting blood glucose measurement. Among these, 8 (29%) fulfilled the diagnostic criteria for diabetes mellitus while 5 (18%) had prediabetes (Table 3).

## 4. Discussion

Finding effective means to prevent T2DM is a critical public health priority. Given the evidence showing that prevention of T2DM with lifestyle intervention is possible [8, 9, 17], there is also an increasing interest in the development of tools to identify high-risk individuals who might benefit from interventions or persons worth further testing for glucose metabolism using the oral glucose tolerance test (OGTT) [18]. However, identification of high-risk subjects, such as those with impaired glucose tolerance, through invasive tests like the OGTT is not feasible at the population level.

The Finnish Diabetes Risk Score can be used as a self-administered test to screen subjects at high risk for T2DM. It can also be used in the general population and clinical practice to identify undetected T2DM, abnormal glucose tolerance, and metabolic syndrome. In our study, almost one-third of those who had a FINDRISC score above 15 were shown to be diabetic and nearly half of them were either prediabetic or diabetic. Our results suggest that FINDRISC is a suitable screening tool for detecting T2DM in a young Arab population. To our knowledge, this is the first study which has investigated diabetes risk factors and the use of FINDRISC in a student population in the Middle East.

In our study, 5.2% of students had a moderate risk and 1.8% had a high risk of T2DM. In a study of the general population in Helsinki, Finland, 66% of men and 70% of women with a moderate or high FINCRISC score turned out to have undiagnosed T2DM [19]. Our study was a population-based study with a large sample size, which is likely to be representative of the student community, which means that a significant proportion of young students in Jordan are likely to develop T2DM.

Elevated blood sugar and impaired glucose tolerance have numerous negative consequences, of which long-term complications such as cardiovascular disease, renal failure, and retinopathy are the most dreaded. Universities might be a suitable arena to promote a healthy lifestyle early in adulthood, for example, by offering health education, healthy food, sport clubs, and other activities. Although targeted interventions among high risk individuals have been shown to prevent development of T2DM [17], public health interventions might have an even stronger impact at the population level [20, 21].

Our study had certain weaknesses. First, as in most diabetes studies, the risk assessment was based both on subjective and on objective assessments. Thus, it is possible that students underreported habits which are perceived as negative, like physical inactivity. Second, we did not measure fasting blood glucose in all participants, and therefore, we were unable to estimate the exact sensitivity and specificity of FINDRISC in this setting. Further studies are needed to validate risk scores for diabetes in a Middle Eastern setting in various age groups. The main strength of this study was the large sample size and the high response rate, which probably means it is representative of the population studied.

In conclusion, we have shown that risk factors for T2DM are common in a young student population in Jordan. The FINDRISC appears to be a suitable tool to identify high-risk individuals in this young age group. The large number of students with overweight and physical inactivity is of particular concern, and public health programs need to initiate preventive interventions early in life in order to turn the epidemic of diabetes in the region.

## Figures and Tables

**Table 1:** Risk factors for diabetes among 1821 students at Hashemite University, Jordan.

Variable	FINDRISC points	Total (%)	Men (%)	Women (%)	*p*
BMI (kg/m^2^)					**<0.001**
<25.0	0	1399 (76.8)	646 (68.7)	753 (85.5)	
25.0–29.9	1	313 (17.2)	204 (21.7)	109 (12.4)	
≥30	3	109 (6.0)	90 (9.6)	19 (2.2)	
Waist circumference (cm; male/female)					**0.008**
<94/80	0	1324 (72.7)	694 (73.8)	630 (71.5)	
94–102/80–88	3	407 (22.4)	189 (20.1)	218 (24.7)	
>102/88	4	90 (4.9)	57 (6.1)	33 (3.7)	
Physical activity					0.087
Yes	0	1046 (57.4)	558 (59.4)	488 (55.4)	
No	2	775 (42.6)	382 (40.6)	393 (44.6)	
Vegetables/fruits					0.344
Yes	0	1048 (57.6)	531 (56.5)	517 (58.7)	
No	1	773 (42.4)	409 (43.5)	364 (41.3)	
Antihypertensive medication					**0.046**
No	0	1799 (98.8)	924 (98.3)	875 (99.3)	
Yes	2	22 (1.2)	16 (1.7)	6 (0.7)	
High blood sugar					0.485
No	0	1751 (96.2)	901 (95.9)	850 (96.5)	
Yes	5	70 (3.8)	39 (4.1)	31 (3.5)	
Relatives with DM					**0.005**
No	0	462 (25.4)	213 (22.7)	249 (28.3)	
Second degree	3	908 (49.9)	471 (50.1)	437 (49.9)	
First degree	5	451 (24.8)	256 (27.2)	195 (22.1)	

BMI: body mass index; DM: diabetes mellitus.

**Table 2:** FINDRISC score among 940 male and 881 female students at Hashemite University, Jordan.

	Risk	Total (%)	Men (%)	Women (%)	*p*
FINDRISC score					0.005
0–6	Low	1218 (66.9)	618 (65.7)	600 (68.1)	
7–11	Slightly elevated	477 (26.2)	242 (25.7)	235 (26.7)	
12–14	Moderate	94 (5.2)	54 (5.7)	40 (4.5)	
15–20	High	32 (1.8)	26 (2.8)	6 (0.7)	

**Table 3:** Fasting plasma glucose in 28 students with a FINDRISC score of 15 and above (high-risk group).

	Plasma glucose (mg/dL)	Number	Proportion (95% CI)
Normal	<100	15	54% (36–70)
Prediabetes	100–125	5	18% (8–36)
Diabetes	≥126	8	29% (15–47)

CI: confidence interval.
